# Qualitative Analysis of Emotional Distress in Cardiac Patients From the Perspectives of Cognitive Behavioral and Metacognitive Theories: Why Might Cognitive Behavioral Therapy Have Limited Benefit, and Might Metacognitive Therapy Be More Effective?

**DOI:** 10.3389/fpsyg.2018.02288

**Published:** 2019-01-04

**Authors:** Rebecca McPhillips, Peter Salmon, Adrian Wells, Peter Fisher

**Affiliations:** ^1^Faculty of Biology, Medicine and Health, School of Psychological Sciences, Manchester Academic Health Science Center, University of Manchester, Manchester, United Kingdom; ^2^Department of Research and Innovation, Greater Manchester Mental Health Trust, Manchester Academic Health Science Center, Manchester, United Kingdom; ^3^Division of Clinical Psychology, Psychological Sciences, University of Liverpool, Liverpool, United Kingdom

**Keywords:** Cognitive behavioral therapy, metacognitive therapy, coronary heart disease, depression, anxiety, qualitative

## Abstract

**Introduction:** Cognitive behavioral therapy (CBT) alleviates emotional distress in mental health settings, but has only modest effects in cardiac patients. Metacognitive therapy (MCT) also alleviates depression and anxiety in mental health settings and is in its initial stages of evaluation for cardiac patients.

**Aim:** Our objective is to compare how CBT and MCT models conceptualize cardiac patients' distress, and to explore why CBT has had limited benefit for cardiac patients and whether MCT has the potential to be more efficacious.

**Method:** Forty-nine cardiac rehabilitation patients, who screened positively for anxiety and/or depression, provided semi-structured interviews. We analyzed transcripts qualitatively to explore the “fit” of patients' accounts of their distress with the main elements of cognitive behavioral and metacognitive theories. Four illustrative cases, representative of the diverse presentations in the broader sample, were analyzed in detail and are presented here.

**Results:** Conceptualizing patients' distress from the perspective of CBT involved applying many distinct categories to describe specific details of patients' talk, particularly the diversity of their concerns and the multiple types of cognitive distortion. It also required distinction between realistic and unrealistic thoughts, which was difficult when thoughts were associated with the risk or consequences of cardiac events. From the perspective of MCT a single category—perseverative negative thinking—was sufficient to understand all this talk, regardless of whether it indicated realistic or unrealistic thoughts, and could also be applied to some talk that did not seem relevant from a CBT perspective.

**Discussion:** Conceptualizing distress from the perspective of CBT presents multiple, diverse therapeutic targets, not all of which a time-limited therapy would be able to address. Given the difficulty of identifying them as unrealistic or not, thoughts about disease, death or disability may not be amenable to classic CBT techniques such as reality testing. MCT proved more parsimonious and, because it did not distinguish between realistic and unrealistic thoughts, might prove a better fit to emotional distress in cardiac patients.

## Introduction

Depression and anxiety are problems commonly associated with cardiovascular disease. A meta-analysis found that generalized anxiety disorder and panic disorder are over twice as prevalent in coronary heart disease (CHD) patients than in the general population, and are comorbid with depressive disorders in approximately 50% of cases, a rate of comorbidity that is similar to community and psychiatric samples (Tully et al., 2016). Reviews of studies of depression in patients with myocardial infarction (MI) found that 20% of patients from eight studies that used structured clinical interviews reached criteria for major depression, and 14 studies that used validated self-report measures identified depression in 40% of MI patients (Carney and Freedland, 2003; Thombs et al., 2006). Emotional distress in this population is linked to poorer quality of life, increased risk of future cardiac events and mortality, increased use of healthcare, and poorer treatment adherence (Frasure-Smith et al., 2000; Bush et al., 2001; Lespérance et al., 2002; Gehi et al., 2005; Goyal et al., 2005; Carney et al., 2008; Yohannes et al., 2010; Dickens et al., 2012; Müller et al., 2012; Tully et al., 2016).

Cognitive Behavioral Therapy (CBT) is widely recommended to treat anxiety and depression (Ballenger et al., 2001; Allgulander et al., 2003; American Psychiatric Association, 2010; National Institute for Health and Care Excellence, 2011, 2016; Bandelow et al., 2014), including in cardiac patients (Lichtman et al., 2008; National Institute for Health and Care Excellence, 2009; Heart Foundation, 2012; Irish Association of Cardiac Rehabilitation, 2013). Its underpinning theory (Table 1) states that: (i) patients experience negative automatic thoughts (NATs) about themselves, the world and the future, which can be triggered by events such as an MI; (ii) NATs reflect patients' negative beliefs about themselves and the world around them (these can indicate deeply entrenched “core beliefs,” or “schemas,” and “intermediate beliefs,” which are more malleable assumptions, attitudes and rules); (iii) cognitive distortions maintain NATs and negative beliefs (Beck, 1964a,b, 1976). CBT therapists help patients to identify and modify NATs and negative beliefs, typically by challenging patients' beliefs in the accuracy of their thoughts, a process termed “cognitive challenging” or “reality testing” (Beck, 1964a,b, 1976; Beck et al., 1979; Beck and Emery, 1985). Since its inception, CBT theory has been extended to incorporate maladaptive behaviors, including avoidance and reassurance seeking, and heightened attention, for example to bodily symptoms (Table 1) (Clarke and Beck, 2010; Beck, 2011; Beck and Haigh, 2014).

**Table 1:** Key elements of the cognitive behavioral and metacognitive models of distress.

**The cognitive behavioral model of distress**
**Negative automatic thoughts (NATs)**—Spontaneous thoughts that occur in everyday cognition e.g., “I will never be able to exercise again.”
**Cognitive distortions**—Errors in thinking, which can include:
**Catastrophizing**—Negative predictions about the future, with no consideration of other possible outcomes, e.g., “my life is over.”
**Overgeneralization**—Reaching a negative conclusion that goes beyond the present situation e.g., “I have a pain in my chest: I am having a heart attack.”
**Dichotomous thinking**—A situation is viewed in only two categories, e.g., “I will never be able to exercise again.”
**Magnification and/or minimization**—When a person or situation is evaluated the negative is magnified and/or the positive is minimized, e.g., “bad things always
happen to me, and good things always happen to other people.”
**Emotional reasoning**—Something is regarded as true because it is felt strongly, evidence to the contrary being ignored, e.g., “I feel like a worthless person; I am a
worthless person.”
**Labeling**—A negative label is assigned to oneself and/or others, without consideration of other evidence, e.g., “I can't run as far as I used to, I am totally useless,”
“the nurse was rude to me today, he is a nasty person.”
**Mind reading**—Belief that others are thinking negatively about oneself, without considering more positive possibilities, e.g., “everyone thinks I am useless.”
**Imperatives**—A fixed idea of how oneself or others should behave and, when these expectations are not met, a negative outcome is overestimated, e.g., “If I had
done things differently I would not have had a heart attack.”
**Discounting the positive**—Positive experiences or qualities do not count, e.g., “the nurse said I am getting stronger, but that's not true, he's just trying to be nice.”
**Negative beliefs**
**Intermediate beliefs**—Rules, attitudes and assumptions that people have about themselves, others and the world around them, that may not be readily perceptible
to a therapist, e.g., “as long as I don't do too much exercise, I will not have another heart attack.”
**Core beliefs**—Concern peoples' beliefs about themselves, others and the world around them, that may not be readily perceptible to a therapist. These beliefs are
deeply entrenched and regarded as absolute truths. At times of psychological distress latent negative core beliefs are activated, e.g., “I am a failure,” “I am unwanted.”
**The metacognitive model of distress**
**Perseverative negative thinking**-Worry and rumination that focus on self-related topics and are difficult to control.
**Worry:** Future orientated, e.g., “what if I have another heart attack.”
**Rumination:** Past orientated, e.g., “why have I had a heart attack, what could I have done differently to prevent it.”
**Metacognitive beliefs-**The theories and beliefs that people have about their own thinking. These may not be readily perceptible to a therapist.
**Positive metacognitive beliefs**—About the usefulness of worry, rumination and threat monitoring, e.g., “worrying about my health will help me to prevent future
illnesses,” “worrying means I care.”
**Negative metacognitive beliefs**—About the uncontrollability of thoughts and their damaging effects on the body and mind, e.g., “I can't help worrying, I can't
control it,” “worrying will damage my health and I will have another heart attack.”

In mental health populations, meta-analyses show that CBT is an efficacious treatment for anxiety and depression when compared to wait-list or a range of comparison conditions, with medium effect sizes of 0.73 and 0.71, respectively (Hofmann and Smits, 2008; Cuijpers et al., 2013). By contrast, meta-analyses of CBT-based interventions in cardiac patients have been disappointing (Linden, 2013). When compared to usual care or comparison conditions, including physical exercise or educational interventions, effect sizes have ranged from 0.12 to 0.44 (Welton et al., 2009; Dickens et al., 2013; Rutledge et al., 2013; Jeyanantham et al., 2017; Reavell et al., 2018). A recently updated Cochrane review assessed the efficacy of psychological interventions in CHD patients compared with usual care (Richards et al., 2017). Although this review did not report effects for CBT specifically, we calculated the effect size across those reviewed studies that evaluated interventions based on CBT and that reported outcomes for depression and/or anxiety (Burgess et al., 1987; Black et al., 1998; Berkman et al., 2003; Claesson et al., 2005; McLaughlin et al., 2005; Michalsen et al., 2005; Koertge et al., 2008; Freedland et al., 2009; Merswelken et al., 2011; Turner et al., 2012, 2014; O'Neil et al., 2015). Again, effect sizes were small: 0.29 and 0.24 for anxiety and depression, respectively. The dominant study in the Cochrane review, and the largest RCT of CBT for cardiac patients yet undertaken, is the ENRICHD study (Writing Committee for the ENRICHD Investigators, 2003). ENRICHD found that, although CBT improved depression when compared to a usual care control condition, albeit with a small effect size of 0.33, these effects were not maintained at follow up. Paradoxically, there was no effect on the primary outcomes of cardiovascular morbidity and mortality, even though further analysis showed that depression was an independent risk factor for mortality (Carney et al., 2003).

Why CBT has had only modest benefit in cardiac patients, particularly when compared to mental health populations, is not clear. Interventions for cardiac patients have sometimes been delivered by physical health practitioners, particularly nurses, not specialized in psychological interventions (e.g., Burgess et al., 1987; Frasure-Smith and Prince, 1989; Van Elderen-Van Kemenade et al., 1994; Cowan et al., 2001), perhaps reflecting the practical difficulties and resource constraints in offering psychological therapy in the context of continuing clinical care. However, it has also been suggested that the defining practice of CBT—reality testing—might be inappropriate in a clinical context where patients' NATs often reflect a reality shaped by morbidity and disability and real risks of sudden and untimely death (Greer et al., 2010; Taylor-Ford, 2014).

A different psychological intervention that has proven effective in mental and physical health settings is metacognitive therapy (MCT) (Wells, 2009). MCT is based on the metacognitive model of psychological distress (Wells and Matthews, 1996; Wells, 2009). In this model (Table 1) psychological disorder is linked to: (i) the cognitive attentional syndrome (CAS), which is characterized by patterns of perseverative negative thinking (worry and rumination) that is extended and difficult to control, threat monitoring, and attempts to control thoughts, for example by thought suppression and reassurance seeking; (ii) underlying metacognitive beliefs that can be conceptually divided into positive and negative beliefs about perseverative thinking. Positive beliefs concern the theme that repetitive negative thinking (e.g., worry) is a helpful way of anticipating problems, whilst negative beliefs concern the theme that such thinking is uncontrollable and dangerous. Whilst the CBT model emphasizes the content of thoughts, MCT emphasizes perseveration of negative thinking as the process maintaining distress. Unlike CBT, the MCT therapist does not challenge the content of perseverative negative thinking or the veracity of beliefs about the self and world. Instead the therapist helps the patient bring perseverative negative thinking under control, and challenges underlying metacognitive beliefs.

In a recent meta-analysis of anxiety and depression treatment trials in mental health populations, MCT was more effective than waitlist control, with a very high effect size of 1.81, and was more effective than CBT, with a large effect size of 0.97 (Normann et al., 2014). Although no randomized controlled trial (RCT) of MCT for psychological distress in physical health populations is yet complete, support for its potential relevance to distressed cardiac rehabilitation (CR) patients comes from evidence of the association between metacognitive beliefs and psychological distress in a range of physical diseases including cancer (Thewes et al., 2013; Cook et al., 2015), Parkinson's disease (Allot et al., 2005; Brown and Fernie, 2015; Fernie et al., 2015) epilepsy (Fisher and Noble, 2017), chronic fatigue syndrome (Maher-Edwards et al., 2011, 2012), fibromyalgia (Kollmann et al., 2016), and diabetes (Purewal and Fisher, 2018). Clinically significant reductions in anxiety and depression in an open trial of MCT for emotional distress in adolescent and young cancer survivors (Fisher et al., 2015) and in a case series in adult cancer survivors (Fisher et al., 2017) add support for the potential relevance of MCT to anxious and/or depressed cardiac patients.

While RCTs are the “gold standard” for establishing the efficacy of psychological interventions, it is now recognized that complementary kinds of research are needed to provide information that RCTs, alone, cannot. In particular, qualitative methods can illuminate the transferability of interventions into real-life (O'Cathain et al., 2013). Specifically, O'Cathain et al. (2013) point to the need for qualitative methods to assess the “fit” between interventions, as idealized in theory and protocols, and the reality of the conditions they are intended to treat. UK Medical Research Council guidance therefore advocates a combination of quantitative and qualitative methods in treatment evaluation, the value of qualitative methods being specifically to help understanding complex causal pathways and generate new theory (Campbell et al., 2000; Moore et al., 2015). Over recent years, qualitative methods have also been used more widely to examine aspects of the “fit” between formal psychological theory and the “lived experience” of patients with physical health problems, including CR patients (Ciechanowski and Katon, 2006; Tang et al., 2009; Angus et al., 2018). In the present study our primary objective was to compare how CBT and MCT models conceptualize cardiac patients' distress, and specifically to explore the “fit” between these models and patients' own accounts of their experience of distress. Our secondary objectives were to identify potential reasons why CBT has low efficacy in this group and to explore whether MCT has the potential to be more efficacious. Our data were interviews in which CR patients described their distress. We addressed these objectives by examining patients' accounts qualitatively from the perspectives of both CBT and MCT, thereby exploring the “fit” between formal theory and patients' experience.

## Methods

### Sample

This research was conducted as part of the UK NIHR funded PATHWAY programme (PATHWAY; RP-PG-1211-20011; Ethical approval from NRES Committee North West, REC reference: 15/NW/0163), a RCT that compares usual CR to CR plus six sessions of Group-MCT delivered by CR practitioners with additional training in MCT (Wells et al., 2018). Inclusion criteria were a score of ≥ 8 on the depression and/or anxiety subscale of the Hospital Anxiety and Depression Scale (HADS) (Zigmond and Snaith, 1983) and meeting CR eligibility criteria (British Association for Cardiovascular Prevention and Rehabilitation, 2012). Upon entry to the PATHWAY programme, patients completed the HADS (to indicate probable clinical levels of distress) and the Impact of Event Scale Revised (IES-R) (to assess symptoms of post-traumatic stress disorder; Weiss, 2007), and a clinical record form which collected socio-demographic information, details of their cardiac condition, any comorbid health conditions, and any past and/or current medication and/or therapies for psychological distress (Table 2). To enable nested qualitative studies, patients who gave or withheld written informed consent to join the trial were asked for written informed consent for qualitative interviews.

**Table 2:** Selected cases.

	**Sex**	**Age**	**Highest level of educational qualification**	**Employment status**	**Marital status**	**Cardiac condition**	**Comorbid health conditions**	**History of treatment for anxiety and/or depression**	HADS A^*^	HADS D^*^	IES-R^**^
Kieran	Male	60–64	University degree	Full time (paid) employment	Married	Coronary heart disease Coronary artery bypass graft surgery 5 weeks before interview	None	No history of emotional problems before cardiac diagnosis. No past or current relevant medication or psychological therapy	3	8	26
Emily	Female	40–44	GCSE or equivalent	Temporary sick leave	Cohabiting	Myocardial infarction	Arthritis	Low mood and suicidal ideation at various times throughout her life No past or current relevant medication or psychological therapy	12	11	43
Paul	Male	45–49	GCSE or equivalent	Unable to work due to long term disability or ill health	Single	Myocardial infarction	Crohn's disease, renal failure, kidney transplant	Low mood and suicide attempt before his cardiac illness Past medication for depression and anxiety; previous psychological therapy and anger management counseling	18	19	64
Mary	Female	50–54	Diploma	Carer	Married	Angina Coronary angioplasty approximately 2 months before interview	Under investigation for skin cancer; being treated for a large cyst in abdomen	Diagnosis of depression in her twenties Current medication for depression; no previous of psychological therapy	14	12	27

* Scores for both the anxiety and depression subscales of the HADS range from 0 to 21. Scores are categorized: 0–7 normal; 8–10 mild; 11–14 moderate; 15–21 severe (Zigmond and Snaith, 1983).

** *Scores for the IES-R range from 0 to 88. Scores are categorized: 0–23 normal; 24–32 PTSD is a clinical concern; 33–88 probable PTSD diagnosis (Creamer et al., 2003; Weiss, 2007)*.

Of the first 79 patients who consented to take part in the RCT, 77 agreed to being contacted about qualitative research. Sixty patients, purposively sampled to include women and men with differing levels of distress and clinical profiles, were contacted by telephone, of whom 46 provided written informed consent to the qualitative study and took part in interviews. Of the first 43 patients who declined consent for the RCT, 15 consented to be contacted about qualitative interviews, all of whom we contacted by telephone, and 3 of whom provided written informed consent to be interviewed. The total number of patients interviewed was 49; 15 were female, and patients ages ranged from 38 to 79, median 57. Most patients described themselves as White British, 2 as Caribbean, 2 as Asian, and 2 as Australasian. Patients' cardiac conditions were MI, adult congenital heart disease, stable heart failure, stable angina, and atrial fibrillation (Supplementary Table 1). Patients were interviewed during CR, but before starting Group-MCT as part of the PATHWAY trial.

### Procedure

RM conducted interviews in a conversational style in patients' homes (*n* = 39), a private office (*n* = 7), where patients received CR (*n* = 2), or in a public café (*n* = 1), as each patient requested. RM's first meeting with each patient was at this interview. No other author met any patient. To begin each interview, RM explained that she was not a therapist, and assured patients that their comments would be anonymized before disclosure to the research team. An interview guide using open questions and prompts facilitated patients' talk; closed questions were used to probe specific points. The pace, sequencing and length of interviews therefore depended on patients. Interviews lasted 29–105 min (mean 58 min). The interview guide was modified in parallel with analysis, to test and develop emerging ideas. Each patient was asked about their emotional experiences since their cardiac event (e.g., “can you tell me how you've been feeling since your heart attack?”), and to describe in detail distress they had experienced including the content of distressing thoughts, the nature of distressing emotions, when they experienced these thoughts and emotions, and how they responded to them (e.g., “what goes through your mind when you are feeling low?,” “what do you do when you feel sad or anxious?”). They were also asked about their understanding of the causes and consequences of their distress, and their thoughts and feelings about their future (e.g., “why do you think you feel like this?,” “what do you feel about the future?”).

Interviews were audio-recorded, transcribed verbatim and pseudo-anonymized. Microsoft Word was used to manage the data during analysis (La Pelle, 2004). In line with recent recommendations, we sought to tailor methods to specific research questions in order to ensure fidelity to the data and utility in achieving research goals (Levitt et al., 2017). Analysis therefore followed three stages. First, inductive analysis first followed a constant comparative approach whereby we identified commonalities and contrasts in how patients described and understood their distress. RM led this analysis, which was tested and refined in regular discussion amongst RM, PF, and PS, who all read the transcripts, and periodically including AW who read selected extracts. Findings from this stage of analysis will be reported separately.

We then returned to the transcripts and re-examined patients' accounts of their distress from the perspectives of the CBT and MCT models. Analysis combined deductive and inductive elements. The main categories of the CBT and MCT models provided an initial template. The inductive element was not to develop those categories, as in the usual approach to template analysis (Crabtree and Miller, 1999), but to understand their “fit” with patients' experience. Our primary criterion for “fit” was parsimony: how simply the models could be related to patients' accounts of their distress. In addition, we were interested in how readily and how extensively each model could be applied across the varied manifestations of patients' distress; that is, how therapeutically “workable” each model would be. We did not apply a checklist, but relied on our interpretation of the patients' subjective experience; that is, we took a stance resembling that of a therapist who seeks to identify aspects of patients' talk with which s/he can engage. Recognizing that the meaning of data in qualitative research is produced jointly by the participant and the interviewer or analyst—just as in therapy it is a product of the therapist and patient in interaction—we emphasized reflexivity; that is, we regarded our own experience of applying the template as data to be discussed and reflected on. When analyzing from the perspective of the CBT model, we focused on identifying distinct NATs, and then categorized the cognitive distortions apparent in these (Beck, 1976). Often, multiple distortions could be identified in the same text but, to simplify presentation, we coded only the most salient one. When analyzing from the perspective of the MCT model, we focused on identifying talk showing perseverative negative thinking—worry or rumination. When we saw possible underlying beliefs—negative beliefs from the perspective of CBT, or metacognitive beliefs from the perspective of MCT—these were also coded. This part of the analysis proceeded iteratively; any text coded as relevant to one model was re-examined from the alternate perspective. RM led this stage of the analysis also, in regular discussions with PS and PF, and periodically with AW. Taking a “disputational” approach, we identified and developed competing interpretations and tested their validity by evidence-based argumentation amongst the group.

Across the sample, we saw several respects in which the perspectives of the CBT and MCT models could be used to conceptualize CR patients' distress differently. We then purposively selected four transcripts reflecting diversity in the presentation and clinical context of distress for detailed analysis to illustrate that divergence (Table 2). We included patients whose distress was linked primarily to their cardiac event, and those whose distress was linked to other health and non-health problems. We also sought diversity in the profile and intensity of distress as indicated by HADS and IES-R scores. Analysis proceeded as for the overall sample, but focusing only on detailed scrutiny of these cases.

The research team brought different perspectives to analysis. RM has experience of research in medical sociology and the psychology of health language and communication, and specifically of qualitative research on patient experiences of physical health care. AW, PF, and PS are clinical psychologists with experience of physical health care: AW has contributed to the development of CBT and is the originator of MCT, and is experienced in treating various psychopathologies in different patient populations using both therapies; PF is experienced in CBT and MCT and in applying MCT to physical health populations; PS has expertise in qualitative analysis of patients' needs and experiences in physical health care, in the development and synthesis of qualitative methods, and in critical analysis of psychological theory in health care.

Table 3 shows data extracts from each transcript and relevant coding. We selected extracts that illustrated the main features of the analysis across the range of each patient's concerns. In Table 3 extracts are shown in the sequence in which they arose in the interview, italicized text provides context for each extract, ellipses are used to indicate omitted talk, and square brackets indicate RM's talk and explanatory comments. Figures in parenthesis in the main text indicate corresponding extracts in Table 3. Pseudonyms are used and identifying details removed to preserve anonymity.

**Table 3:** Cardiac patients' emotional distress conceptualized from the perspectives of cognitive behavioral and metacognitive models of distress.

**Patients' distress conceptualized from a CBT perspective**			**Patients' distress conceptualized from a MCT perspective**	
**Bold** text shows elements of patients' accounts that a CBT therapist might engage with			Underlined text shows elements of patients' accounts that a MCT therapist might engage with	
Possible negative beliefs	Cognitive distortions contained in negative automatic thoughts		Perseverative negative thinking: worry or rumination	Possible metacognitive beliefs
**Case 1: Kieran**				
		*At the start of the interview, after being asked how he came to be in CR, Kieran recounted events leading to his diagnosis, the diagnostic tests he had undergone and his decision to undergo CABG. RM asked how Kieran felt during the period of diagnostic testing and since CABG:* **1a**.		
		Embarrassed to be honest… somebody says “go on that treadmill” and then after two minutes, “sorry I'm going to have to stop.” **You think****“it**		
“I did not perform as I should, so I am inadequate”	Imperative	**shouldn't be like this, I should be able to go on forever”**… it was a bit disappointing, the gradual realization that your health is not as good as you thought it might have been.	Rumination	
		*Earlier, Kieran had talked about feeling* “sorry for” *himself; RM now prompted him about this*: **1b**.		
		You explain to people what you've been through, you get a bit tearful and you think “oh well, stop feeling sorry for yourself”… snap out of it… you look at other people of a similar	Rumination	
	Overgeneralization	age… they seem to be a lot fitter than you… I was always fit, all my life, and now**I am the most unfit person I know…**		
		*RM prompted him about his feeling that having coronary heart disease felt unfair:* **1c**		
	Magnification and minimization	I've worked hard all my life and you see other guys that**you think haven't worked hard all their lives and they haven't gone to the gym… I get myself reasonably fit and I can't understand, me and them, and they're enjoying themselves.**	Rumination	
		*While responding to a later question about the time that he spent worrying, Kieran introduced talk about his past, and became tearful*: **1d**.		
		You start to think back to the friends that you had… when you're playing old music and then your memories start flooding back and it can be quite difficult… I've deliberately not done that anymore… you work hard and you're getting toward something when you can perhaps start to slow down and you think	Rumination	
“My life would have been better if I had made different choices”	Discounting the positive	“I've got a health problem” and you think “oh where's all my friends,” you left them behind thirty years ago… you move away from your friends and you think**“it wasn't worth it.”**	Rumination	
		*RM asked whether he could ever stop feeling sorry for himself*: **1e**.		
“Retirement will not be as enjoyable as I thought”	Catastrophizing	Only by doing some sort of activity… if you've got a bit more variety in your activities, you're not going to get bored, you don't then sit down and start dwelling on things that might upset you… the last 5 weeks have been a bit of an eye opener… as you approach retirement you think “I actually quite fancy retiring”… but in this last 5 weeks,**this is what retirement is about, I've got to keep working, thank you**… your mind would be more active, you can't always be thinking “what can I do now?”	Rumination Worry	“Thinking is uncontrollable”
**Case 2: Emily**				
		*When RM began by asking how she came to be in CR, Emily recounted in detail the evening of her MI, and the treatment that she had received since. RM asked how Emily had felt since the MI:* **2a**.		
“I am going to have another MI; if I do, I will die”	Catastrophizing	I can't get to sleep… **I think if I go to sleep I'm not going to wake up**… I don't even think about it [heart attack] until I go to bed… [Then] I just re-run everything through my mind, I just re-run that whole Friday night and what if, what if my heart had stopped beating before we'd got to [the hospital]	Worry Rumination	“Thinking will help me to find answers”
		*RM prompted Emily about her fear that she might not wake up:* **2b**.		
“If I have another MI, I will die”	Catastrophizing	The heart attack to me was not like you would see a heart attack on the TV… the grabbing the chest… you drop to the floor… it wasn't that bad, so in my head… the pain that I had when I had my heart attack, I could sleep through that… I think that's where	Rumination	
		the worry comes from, because**if the same thing happens again, I would probably stay asleep, or what if the stent doesn't hold out**… it's all these things going through my head, because it's only a thin little piece of metal”	Worry	“Thinking will help me to be prepared”
		*After talking about avoiding going to bed due to her fear of not waking up, and about not wishing to disclose her fears in the context of CR, Emily recounted discussing her MI with a friend who had undergone CABG*: **2c**.		
“I am stupid”	Emotional reasoning	This is why I think **I feel stupid** because he's [friend] had a double heart bypass, I've just had a stent	Rumination	
		put in, it's nothing… I rang him up I said “has it affected you,” and he was like “no… just carrying on with life”… and there's me thinking shit, well why has it affected me, all I've got is a STENT”	Rumination	“Thinking will help me to find answers”
		*RM prompted Emily to talk more about concerns that she had mentioned earlier, first about her sleep:* **2d**.		
	Catastrophizing	**I worry about not being able to sleep when I go back to work**,because that would worry me because I need to be able to sleep, so that I can do my job correctly”	Worry	
		*Then about why she continued to think about the night she had her MI*: **2e**.		
	Catastrophizing	I lie in bed thinking “stop bloody thinking about it… you're alive for god's sake”… I'm tossing and turning but it's still always there, in the back of my mind”… no matter how many times I run through it… everything that's been said to me… I think the problem is I've got a medical background… I know a bit too much and that doesn't help me at all… it makes it worse”… [RM: Is it every night you are like this?] “Yeah… there have been nights where I've not slept at all… so in the day time I have fallen asleep on the sofa…**in my head it's okay to sleep in the day because somebody would find me**.	Rumination Worry	‘‘Thinking is uncontrollable”;“thinking will help me find answers”; “thinking is harmful”
**Case 3: Paul**				
		*When asked how he came to be in CR, Paul talked about delays in responding to his MI because of GP misdiagnosis, and about this resulting in a worse prognosis than had he been treated for his MI sooner. He also spoke about his Crohn's and kidney diseases. When recounting his kidney transplant, he introduced his feelings about an ex-partner, from whom he had separated 2 years previously. He said he still thinks about their break-up daily. He said that he keeps busy to avoid thinking about this and other concerns and explained:* **3a**.		
		But now I'm not working and I've got more time on my hands, I'm my own worst enemy because… the only time I'm alright is when I'm asleep, because then I am not thinking about things… I analyse everything… I'm terrible.”	Rumination	“Thinking is uncontrollable”
		*Paul explained that he “analyses everything”; and talked again about his previous relationship*: **3b**.		
“It is all my fault”	Imperatives	I start analyzing it [past relationship]… I'm trying to assert blame… who's at fault…**is it something I could have foreseen and maybe done something about**… it's that “if only” scenario when I sit there thinking	Rumination	“Thinking will help me find answers”
		**“well if only I'd done that, or if only I hadn't said that, if only I'd gone about it this way'**… it just goes round and round and round up there… it's easier to blame yourself than it is to blame others”	Rumination	“Thinking will help me find answers”
		*RM prompted Paul about how much he blamed himself:* **3c**.		
“It is all my fault”	Imperative	“Quite a lot really… this you know, could I have foreseen the fact that I was going to have a heart attack,**I blame myself for not ringing an ambulance when I should have done**… maybe things would have been different, but then if I think like that it's just going to eat away, but it does… I think “god all I had to do is phone nine nine nine… but then I felt alright on Sunday… so you think to yourself “oh I must be alright.”	Rumination	“Thinking will help me find answers”
		*While discussing how Paul keeps busy to stop thinking like this, he introduced his childhood:* **3d**.		
“I expect that bad things will always happen to me”	Catastrophizing	My childhood hasn't been brilliant… the relationship with my dad is not very good, he was a bit of a tyrant to say the least…**I expect [bad] things to happen because that's the way I sort of see it… if it's going to happen, it's going to happen to me**… but it's that analyzing again… if it had been anybody else they wouldn't have probably lost their job over it, whereas I have.	Rumination	“Thinking will help me find answers”
	Dichotomous thinking	**I can't drive a truck anymore, so that's my career gone… I can no longer afford to stay living in me flat… so it's not just a matter of having a heart attack… [another person who has had a heart attack] they've got a partner who they can rely on, and you're just thinking “why me”**		
		*RM asked Paul how often he thinks about the problems he had told her about:* **3e**.		
“I am helpless”	Imperative	All the time really… even when I was at work… me mind is ticking away with things all the time… you get home and you just don't seem to be able to switch off.” [RM: How long have you been thinking about these things?]… Oh years… as far back as I can remember to be honest… because it wasn't good with my Dad…**if I'd have probably been brought up or treated differently… these things might not have happened.**	Rumination	“Thinking is uncontrollable”
		*RM then asked Paul how he copes with feeling low:* **3f**.		
		I just want to be on my own… I don't want to bump into someone and then I know what they are going to say, “are you alright”… and you've got to go		
	Mind reading	“yeah, of course I am… how are you?” And then they start going on about their troubles… I'm thinking to myself,**“you don't know the flaming half of it”**… that just makes me feel worse… angry… they're going on about how they've stubbed their toe on a door… these people,**there is nothing**	Rumination	
	Magnification and minimization	**wrong with them… and you're just thinking I'll swap with you tomorrow… you don't know the half of it'…**“I know it's definitely not useful [to think this way], I know it's more harmful than good, I know that… but I still do it.”		“Thinking is harmful”
**Case 4: Mary**				
		*When Mary discussed what led to her admission to CR at the start of the interview, she talked, not only about her angina, but also about other physical health conditions and about being diagnosed with depression in her twenties, resulting in her taking anti-depressant medication ever since. Mary explained:* **4a**.		
	Catastrophizing	“That [physical health conditions] did all did get me down, and made me cry… I just felt bombarded with hospital appointments…all that plays on my mind… with my work as well…**maybe not being able to look after**[adult Mary cares for]… so that worries me, putting him somewhere… [I am] worrying about going in and having the stuff [operations] done.”	Worry	
		*When RM asked Mary to describe her feelings about this impending treatment, she spontaneously introduced her relationship with her husband*: **4b**.		
“I am worthless” “I am worthless”	Emotional reasoning Emotional reasoning	“Me and my husband are going through a not good patch… just loads of different things which really, really get me down… it's just what goes on in my head…**I feel like a worthless person…**and my husband makes me feel like that” [Mary becomes tearful]… [RM: Why do you feel worthless?] Because I feel like I am made to feel like that…**my opinion or anything I say don't mean anything…**I feel like it's not worth listening to”	Rumination	
		*Earlier, Mary had mentioned her fears for her family, mainly for her son whose alcohol consumption concerned her, and for her relationship with her husband. RM now prompted Mary about those concerns:* **4c**.		
	Catastrophizing	It's mainly [my son], I just want him right… I can't get on with my own life… I can't see further than the day…**I can't see a future or anything…**because I worry about when he's [husband] coming home from work, what	Worry	
		he's going to say, I'm just on pins constantly… he [husband] thinks he is the most marvelous person going		
		*RM asked Mary if she was concerned about her cardiac health; Mary responded that she was not, and then spontaneously returned to how she felt about herself:* **4d**.		
“I am ugly”	Labeling	I mean I don't like myself,**I think I am ugly…**I've got low self-esteem about myself, I can't even speak right… [RM: How long have you been feeling like this for?]… “A long time… maybe a couple of years I have been feeling like this… I mean I won't even go out, I go shopping and I go to my sister's to get dressed up,**I just think everyone's thinking**	Rumination	
“I am worthless”	Mind reading Emotional reasoning	**what a fat ugly cow”** [RM: How often do you think these thoughts?] every day… if I put make up on, someone will say “oh you look pretty,” I just think they are lying… my sister, she'll say “put a bit of make up on, you'll feel better,” but because**I feel worthless**I think what's the point.		
		*Mary talked more about her concerns for her relationship and her son:* **4e**.		
		All I want is my family to be right… all I want is to be happy and I'm just not [crying]… [RM: so do you spend a lot of time worrying about your family and thinking about all these things?] All the time… the minute I wake up and the minute I go to sleep… eighty per cent [of the time… thinking about]… if he's [son] at his flat, I'm worrying myself sick, if he's drinking…**when he don't answer his phone, I think he's dead”**…	Rumination	“Thinking is uncontrollable”
“If my son drinks, the worst will happen” “If my son drinks, the worst will happen”	Catastrophizing Catastrophizing	**thinking he's drank himself into a stupor and choked and died…**and how am I going to get into his flat, I mean I've been to hospital ten times with him… he broke his jaw twice… the first time I ever went to hospital with him, he ended up having a seizure…**every time he drinks, I think he is going to have a fit**… I'm just worried sick about him all the time.	Worry	“Thinking will help me to be prepared”
		*Mary explains that she talks to her husband about her concerns for her son:* **4f**.		
		My husband seems to dismiss it [concern for her son] and says “oh forget about it, get on with your life”… how can I, what mother would I be?'… I'm his mother and mothers do worry about their kids… especially when it's something like this	Worry	“Thinking is uncontrollable”
		[RM: so would you say you can't help it?] I can't help it, no… if I knew he was alright I'd be happier… I can probably over-analyse a lot of stuff… dealing with something in my head that hasn't happened yet, but maybe it will… I'm sat here worrying myself sick over him… I just can't help it, I can't switch off from it.”	Worry	“Thinking will help me to be prepared”

## Results

As will be reported elsewhere, inductive analysis of all interview transcripts identified CR patients' diverse concerns about their cardiac event, including the continuing risk to their lives and the constraints that reduction in energy or confidence, as well as continuing medical treatment or surveillance, imposed on their lives. Patients also reported dwelling on regrets from their early life and worrying about current or future challenges unrelated to their physical health.

When considering the transcripts from the perspectives of CBT or MCT, CR patients' distress was conceptualized very differently. From the perspective of CBT, we identified relatively focused and distinct sections of patients' talk that contained NATs, and that displayed many cognitive distortions (Beck, 1976), although it was often difficult to agree which category of distortion to allocate specific NATs to. It was also sometimes difficult to decide whether specific negative thoughts, particularly relating to cardiac disease, were realistic or unrealistic. We were also able to identify patients' negative beliefs about themselves, (such as being inadequate), about the world around them (such as other people seeing them as inadequate), and about the future (for example that life is pointless). From the perspective of MCT, we identified longer sections of patients' talk, which could readily be understood as perseverative negative thinking (worry or rumination), and which encompassed many of the different NATs and cognitive distortions that were categorized in detail from the CBT perspective. These chains of perseverative negative thinking included some talk that had not been coded as relevant from a CBT perspective. We also identified patients' positive metacognitive beliefs, including that rumination would help them understand why they had experienced a cardiac event and that worry would prepare them for a future one. We identified negative metacognitive beliefs, also, specifically that thinking about illness was uncontrollable and harmful and threatened their mental health or risked worsening their cardiac disease.

Table 2 describes the four cases selected to develop and illustrate these findings. Kieran, with HADS scores suggesting mild depression, described no history of emotional problems before his diagnosis of CHD and subsequent coronary artery bypass graft (CABG). He attributed his unhappiness mainly to his cardiac illness and the threat it presented to enjoying his impending retirement. Emily, with HADS scores suggesting moderate anxiety and depression, had felt depressed and even suicidal before her MI, although she had never been diagnosed with anxiety or depression. Her concerns centered on fear of another MI and difficulty sleeping. Paul, whose HADS scores suggested moderate anxiety and depression, had been diagnosed with depression and anxiety, had attempted suicide previously, and had received psychotherapy and anti-depressant medication. In addition to concerns about the loss of his career, and therefore his home, because of his MI, Paul dwelled on the breakdown of previous romantic relationships and on difficulties in his relationship with his father during childhood. Mary had been diagnosed with angina and treated with a coronary angioplasty. Her HADS scores suggested moderate anxiety and depression. She had a history of depression, and still took anti-depressant medication. Her concerns centered on her son's alcoholism and her problematic relationship with her husband.

### Conceptualizing Patients' Distress From a Cognitive Behavioral Perspective

Each patient's talk showed a diverse range of NATs concerning fear of their cardiac disease and the impact this already had on their lives, or may have in future. Paul (3b,d,e) and Mary (4b,c,e,f) also voiced NATs about social and family relationships, and Kieran (1d) and Paul (3b,c,d,e) about past events. Every patient's talk also indicated possible negative beliefs. Like their NATs, these were diverse. Some were specifically related to cardiac disease. For instance, Kieran (1d,e) described believing that his retirement would be less fulfilling than he had imagined, because of limitations he envisaged as a result of his CHD, Emily (2a,b) indicated the belief that she would have another MI that would be fatal, Mary (4a) was concerned that she would be unable to fulfill her role as a carer, and Paul (3c,d) believed that the effects of his MI on his health might have been mitigated had he called an ambulance sooner, and treated earlier. However, many negative beliefs concerned other areas of life, even pre-dating cardiac disease. Mary (4b,c,d) talked openly about being “stupid,” “ugly,” and “worthless,” indicating persistent negative beliefs about herself that she described having for “years,” before being diagnosed with angina. Paul (3b,d) blamed himself for neglecting previous romantic relationships, and for not seeking medical help for his MI earlier than he did. Additionally, Paul (3e) described consistently expecting “bad things” to happen to him that he was helpless to influence, such as breakdown in relationships, complaining that he might have been able to prevent them had he been “brought up or treated differently.”

Every patient displayed several cognitive distortions (Beck, 1976). Catastrophizing was common, particularly in NATs concerning cardiac disease: for example, Emily's NATs (2a,b,d,e) that she would have a second MI, and that this would kill her, and Kieran's (1e) NAT that his CHD would devastate his retirement plans. For Paul and Mary, catastrophic thinking extended beyond cardiac disease, for instance in Paul's (3d) NAT that “bad things” happen to him and Mary's (4e) NAT that that her son would die if he returned to drinking alcohol. Patients' NATs contained further distortions, including magnification and minimization, imperatives, dichotomous thinking, and emotional reasoning. For example, Kieran's (1c) NATs contrasting his own experiences with others' illustrated magnification and minimization: he minimized others work and efforts to be healthy when complaining that “they haven't worked hard all their lives and they haven't gone to the gym,” and magnified their experiences when he suggested “they're enjoying themselves.” Paul's (3b,c) NATs concerning previous relationships and his MI contained imperatives: he lamented that “if only” he had done things differently his relationship might not have broken down, and that he “should” have called an ambulance sooner, and his NATs (3d) concerning the impact of his MI contained dichotomous thinking: that he “can't drive a truck,” and that his “career” [had] “gone.” Emily and Mary displayed NATs that contained emotional reasoning, as Emily (2c) described “feel[ing] stupid” because her STENT had “affected” her, in contrast to a friend who had been unaffected by a double heart bypass, and Mary (4b) “[felt] like a worthless person,” because her husband made her feel that “anything [she said] don't mean anything.”

Some of the NATs, negative beliefs and cognitive distortions described above seemed clearly unrealistic, for example Kieran's (1a) minimization and magnification. However, others reflected elements of patients' clinical reality. Because of the physical impact of his MI, Paul (3d) could no longer drive a truck, and so had lost his job and could not afford to keep his flat. Mary's (4a) concerns about not being able to work as a carer predict a likely, albeit temporary, consequence of her clinical reality. In many instances, however, it was difficult to categorize the NATs, negative beliefs or cognitive distortions as either realistic or unrealistic. For instance, Kieran's (1e) prediction that his CHD would preclude activities he had planned for retirement might reflect the reality of disability associated with CHD for many patients, and Emily's (2a,b) fear of a fatal MI might reflect a real, albeit potentially small, risk.

We coded only the most salient cognitive distortion for any NAT. However, multiple distortions could readily be identified in many NATs, and it was often difficult to decide which to prioritize. For instance, Kieran's (1b) NAT about being “the most unfit person I know' shows over-generalization, but might also be regarded as magnification and minimization or as labeling. Similarly, we coded Mary's (4e) NAT that, if her son drank alcohol again, it would kill him as catastrophic thinking, but it also might be understood as overgeneralization or dichotomous thinking.

In summary, each patient's distress could be readily conceptualized using the CBT model. This involved identifying a diverse range of NATs, negative beliefs and cognitive distortions. However, distinguishing between unrealistic and realistic NATs and negative beliefs could be difficult, particularly when they were associated with patients' cardiac disease.

### Conceptualizing Patients' Distress From a Metacognitive Perspective

Every patient described experiences that could readily be interpreted as indicating perseverative negative thinking. For example, Paul (3a) explained that the only time that he did not ruminate was while asleep, Emily (2e) complained that rumination about the night of her MI prevented her from sleeping, and Mary (4e) reported being “worried sick” about her son “all the time,” all clearly indicating the repetitive and extended thinking that characterize perseverative negative thinking. Every patient also displayed perseverative negative thinking in “real time” during their interviews. For instance, Mary repeatedly talked about her son's drinking, her relationship with her husband, and about how she felt about herself, even when asked about seemingly unrelated topics. Similarly, Paul introduced regrets over his previous relationships and childhood repeatedly throughout his interview. The single category of perseverative negative thinking encompassed most of the talk that, from the perspective of CBT, was understood as diverse NATs, negative beliefs, and cognitive distortions. It also included sections of talk that were not coded as significant from the CBT perspective, for example Kieran's (1d) regrets about losing contact with old friends, Emily's (2e) description of mentally “re-running” her MI, Paul's (3a) explanation that the only time he is “alright” is when asleep, and Mary's (4f) account of “worrying” about her adult children, particularly her alcoholic son.

Each patient's account contained evidence of negative metacognitive beliefs (Wells, 1995). Kieran (1e) alluded to believing that he lacked control over thinking, when he described needing to keep busy during retirement so that he would not “always be thinking.” The other patients were more explicit about believing they lacked control. Mary (4f) and Paul (3e) reported being unable to “switch off” worrying or ruminating, and Emily (2e) described rumination that was “always there.” Emily and Paul also indicated possible beliefs about the harmfulness of thinking. Emily (2e) referred to her “medical background” as a “problem” because knowing “too much” made her “worse” (2e), while Paul (3f) explained that “thinking to [him]self” about the differences between himself and others was “more harmful than good.” Three patients' accounts also demonstrated positive metacognitive beliefs that thinking would help them to find answers and be prepared. Emily (2a,c,e) reported “re-running” the night of her MI and continuing to ask herself why her MI had affected her so badly. Similarly, Paul (3b) described “analyzing” a past relationship, lest he could have “foreseen and maybe done something about” its breakdown, and Mary (4e) explained that she deals “with something in [her] head that hasn't happened yet, but maybe it will.” Much of the text coded as metacognitive beliefs from the perspective of MCT was not coded at all from the perspective of CBT; for example text coded as Emily's (2a,e) metacognitive beliefs that thinking about the events that led to her MI was uncontrollable and harmful but would help her find answers, and as Mary's (4f) metacognitive beliefs that worrying is both uncontrollable and useful.

In summary, conceptualizing cardiac patients' distress from the perspective of MCT proved more parsimonious than from that of CBT; the diverse range of NATs, cognitive distortions and negative beliefs identified from the perspective of CBT were largely contained within the single category of perseverative negative thinking, which also included text not coded as relevant to CBT. There were possible metacognitive beliefs, also often in text not coded as relevant to CBT. Examining accounts from the perspective of MCT did not require decisions as to whether distressing thoughts were unrealistic.

## Discussion

We addressed our primary objective by comparing how CR patients' accounts of distress can be understood from CBT and MCT perspectives. Focusing on the content of patients' concerns, taking a CBT perspective meant allocating specific elements of patients' accounts of their distress across many discrete categories: different NATs and negative beliefs and the multiple cognitive distortions that maintain them. In contrast, MCT was more parsimonious in that a single category—perseverative negative thinking—encompassed most of that text. Moreover, understanding distress from the perspective of CBT involved difficult judgments as to whether negative thoughts were realistic or unrealistic, whereas this distinction was not necessary from an MCT perspective. Our findings therefore address our secondary objectives in pointing to potential reasons why CBT has low efficacy for cardiac patients and why MCT might have a better fit with distress in this population.

Our findings suggest two respects in which the CBT model might be a poor fit with CR patients' distress. First, patients described diverse NATs and negative beliefs, encompassing not only cardiac disease but other areas of their lives including problems that long pre-dated their cardiac diagnosis. In the context of a time-limited therapy, it will rarely be possible for a CBT therapist to address all these for any patient. CBT therapists are advised to target specific NATs and negative beliefs according to how distressing or dysfunctional they are, or how persistent they are (Beck, 1976). However, CBT theory does not itself specify how a therapist can choose between multiple NATs and negative beliefs that might all be distressing, dysfunctional and persistent, and does not stipulate how addressing specific NATs and negative beliefs will alleviate distress caused by unaddressed NATs and negative beliefs. Furthermore, since a single NAT can contain more than one cognitive distortion, it can be difficult to decide which is the “main” one, leading to the risk that the CBT therapist will not target the most critical distortion.

The second reason why the CBT model had questionable fit with CR patients' distress was because of the importance it attaches to identifying and challenging unrealistic thoughts and beliefs. In the context of a recent life-threatening cardiac event, continuing vulnerability to cardiac problems, and attendant disability and uncertainty about the future, patients had many negative thoughts and beliefs that could not be readily categorized as realistic or unrealistic. Reality testing patients' realistic concerns about their health, and about the toll that ill-health has taken, or might take, on their lives is, at best, inappropriate and, at worst, absurd (Greer et al., 2010). CBT theory has therefore evolved to address the challenge of realistic negative thoughts in the context of life-threatening disease, particularly cancer (Greer et al., 2010; Moorey and Greer, 2012; van de Wal et al., 2015; Temple, 2017). However, adaptations of the theory center on differentiating between realistic and unrealistic concerns; realistic concerns can then be targeted using problem solving. Trying to decide whether NATs and negative beliefs are realistic or not, particularly in the context of real disability, and small but potentially fatal risks of further cardiac events, adds further complexity to the therapeutic process. Moreover, engaging patients in such decisions risks exacerbating their distress—for instance where they focus on a small, but real chance of a further MI in the future.

MCT requires fewer difficult or arbitrary decisions from a therapist. It is not necessary to identify the negative thoughts that are “most important,” or to distinguish realistic from unrealistic ones. Instead, an MCT therapist would understand the diverse concerns of CR patients as evidence of a single process of perseverative negative thinking. Moreover, elements of patients' accounts that cannot be understood in terms of any of the classic categories of the CBT model are significant from the perspective of MCT when they are further examples of perseverative negative thinking. Our findings also showed that patients make their metacognitive beliefs, which, according to the MCT model, maintain perseverative negative thinking, accessible to therapists. MCT might therefore have a better fit with distress in CR patients, because MCT therapists can target perseverative negative thinking, and the metacognitive beliefs that underlie this, and thereby address patients' diverse concerns, both unrealistic and realistic, in a time-limited therapy.

## Limitations

As a qualitative study in a single setting, our findings cannot automatically be generalized. However, our qualitative approach allowed us to explore the “fit” between theory and reality in a way that is grounded in patients' own accounts of their distress. The selection of only four patients' transcripts for detailed analysis risks what Onwuegbuzie and Leech (2007) call “key informant bias” if those accounts do not represent the full sample. To minimize this risk, we selected cases across the range of severity of distress and diversity of concerns seen in the whole sample. Moreover, these patients' concerns resembled those seen in previous qualitative reports of cardiac patients' distress (Jensen and Petersson, 2003; Andersen et al., 2008; Dekker, 2014). Qualitative research is necessarily shaped by researchers' own perspectives in both data collection and analysis phases. Two members of the research team, while expert in both MCT and CBT, have been proponents of MCT. However, by including authors with experience in using qualitative methods to voice patients' own perspectives and challenge idealized theories, and by adopting an iterative, “disputational” approach to analysis, we sought to ensure an analysis that was thoroughly tested and informed from multiple perspectives while being grounded in patients' accounts.

## Conclusions

A strength of this study is the combination of inductive and deductive analysis. By using CBT and MCT as templates through which to view CR patients' emotional distress, we have been able to explore the fit between patients' distress and these two therapeutic modalities. The findings help to understand the limited efficacy of CBT in cardiac patients, and suggest that MCT might offer a better fit with distress in this population. Both on scientific grounds, and to reduce the risk of wasting resources in evaluating interventions that are a poor fit to the needs of the target population, we suggest that studies such as this should be prioritized early in the development and evaluation of psychological interventions, or in the application of interventions to new populations, before progressing to the stage of RCTs. Such studies can ensure that RCTs are focused in areas of the greatest potential benefit to patients. More specifically, our findings can inform improvements in psychological intervention in the context of cardiac care. We propose that, rather than conducting further RCTs of CBT for cardiac patients, evaluations of potentially more compatible psychological interventions such as MCT are required.

## Author Contributions

RM collected the data and led the data analysis of each transcript in its entirety. PF and PS contributed to the data analysis in its entirety. AW contributed to the data analysis of selected extracts from specific transcripts. RM led the development of the manuscript. PF, PS contributed to the first and subsequent drafts of the manuscript. AW contributed to later drafts of the manuscript.

### Conflict of Interest Statement

AW is a co-director of the MCT Institute. The remaining authors declare that the research was conducted in the absence of any commercial or financial relationships that could be construed as a potential conflict of interest.

## Abbreviations

CABG: Coronary artery bypass graft
CAS: Cognitive attentional syndrome
CBT: Cognitive behavioral therapy
CHD: Coronary heart disease
CR: Cardiac rehabilitation
HADS: Hospital anxiety and depression scale
IES-R: Impact of event scale—revised
MCT: Metacognitive therapy
MI: Myocardial infarction
NAT: Negative automatic thought
NICE: National Institute for Health and Care Excellence
RCT: Randomized controlled trial.
