# Supplementary material for: Qualitative Analysis of Emotional Distress in Cardiac Patients From the Perspectives of Cognitive Behavioral and Metacognitive Theories: Why Might Cognitive Behavioral Therapy Have Limited Benefit, and Might Metacognitive Therapy Be More Effective?
Source: Front Psychol. 2019 Jan 4;9:2288. doi: 10.3389/fpsyg.2018.02288 (PMC6328488; doi:10.3389/fpsyg.2018.02288)
Supplement: Supplementary file 1 [file Table_1.pdf]

Supplementary table 1: sample characteristics

[illegible]dentification [illegible]umber	Gender	Age range	Ethnicity	Highest level of educational qualification	Employment status	Relationship Status	Cardiac condition	Comorbid health conditions	History of treatment for anxiety and/or depression	HADS scores*	IES[illegible] scor[illegible]
C01	Female	45-54	Caribbean	GCSE or equivalent	In full time (paid) employment	Cohabiting	Acute coronary syndrome (myocardial infarction)	Hypertension; arthritis	No history of relevant medication; has received CBT previously	A=9 D=3	21
[illegible]02	Male	45-54	White British	GCSE or equivalent	In full time (paid) employment	Married	Acute coronary syndrome (myocardial infarction)	Hypertension; high cholesterol	No history of relevant medication or psychological therapy	A=10 D=3	45
[illegible]03	Female	65-74	White British	Vocational qualification	Retired	Divorced	Adult congenital heart disease	Hypertension; diabetes type 2; MS; COPD; arthritis; chronic fatigue; fibromyalgia; high cholesterol	Past medication for anxiety; current medication for depression; has received counselling previously	A=14 D=10	47
C04	Male	45-54	White British	Diploma	In full time (paid) employment	Cohabiting	Acute coronary syndrome (myocardial infarction)	High cholesterol	Past medication for depression; has received psychological therapy previously (therapy type	A=8 D=3	33

									not disclosed)		
[illegible]05	Male	75+	White British	Not collected	Not collected	Not collected	Not collected	Not collected	Not collected	Not collected	Not coll[illegible]
[illegible]06	Female	65-74	White British	None	Retired	Married	Acute coronary syndrome (myocardial infarction)	Hypertension; atherosclerosis; COPD; arthritis; incontinence	Current medication for depression; has never received psychological therapy	A=8 D=8	29
C07	Male	55-64	White British	None	Unemployed	Separated	Acute coronary syndrome (myocardial infarction)	Hypertension; high cholesterol; COPD; arthritis; urethral trauma	No history of relevant medication or psychological therapy	A=10 D=5	85
C08	Male	45-54	Asian	Diploma	Unemployed	Married	Acute coronary syndrome (myocardial infarction)	Hypertension; diabetes type 2; high cholesterol; atherosclerosis; COPD; hernia	No history of relevant medication or psychological therapy	A=9 D=9	29
C09	Male	75+	White British	None	Retired	Widowed	Stable heart failure	Hypertension; diabetes type 2; arthritis	No history of relevant medication or psychological therapy	A=2 D=13	1
[illegible]10	Male	45-54	White British	None	Unable to work due to long term disability or ill health	Divorced	Acute coronary syndrome; Stable heart failure	Hypertension; high cholesterol; COPD	No history of relevant medication or psychological therapy	A=5 D=12	21
[illegible]11	Male	55-64	White	GCSE	Unable to	Divorced	Adult	Hypertension	No history of	A=10	5

			British		work due to long term disability or ill health		congenital heart disease		relevant medication or psychological therapy	D=10	
[illegible]12	Female	55-64	White British	Postgraduate degree	Retired	Single	Acute coronary syndrome (myocardial infarction)	COPD; IBS	Current medication for depression and anxiety; has received person-centred therapy and mindfulness therapy previously	A=14 D=11	41
[illegible]13	Male	45-54	Australian	Postgraduate degree	In full time (paid) employment	Separated	Acute coronary syndrome (myocardial infarction)	Hypertension; epilepsy	No history of relevant medication or psychological therapy	A=10 D=8	39
[illegible]14	Male	45-54	White British	GCSE	Unemployed	Separated	Acute coronary syndrome (myocardial infarction)	COPD	No history of relevant medication or psychological therapy	A=7 D=9	24
[illegible]15	Male	55-64	White British	None	In full time (paid) employment	Married	Adult congenital heart disease	Hypertension; COPD	No history of relevant medication or psychological therapy	A=15 D=8	10
[illegible]16	Male	55-64	White British	None	Retired	Married	Acute coronary syndrome	Hypertension; high cholesterol; IBS	Current medication for depression and	A=15 D=11	48

							(myocardial infarction)		anxiety; has received counselling previously		
**[illegible]17*** **	**Male**	**55-64**	**White British**	**Degree**	**In full time (paid) employment**	**Married**	**Coronary heart disease**	**None**	**No history of relevant medication or psychological therapy**	**A=3 D=8**	**26**
C18	Male	45-54	Caribbean	A level	In part time (paid) employment	Single	Acute coronary syndrome (myocardial infarction)	Hypertension; diabetes type 2; high cholesterol; atherosclerosis; partially sighted	No history of relevant medication or psychological therapy	A=12 D=5	56
[illegible]19	Male	35-44	Duel White British and New Zealand	Postgraduate degree	In full time (paid) employment	Cohabiting	Adult congenital heart disease	Hypertension	No history of relevant medication; has received counselling previously	A=8 D=5	55
C20	Female	65-74	White British	None	Retired	Divorced	Acute coronary syndrome (myocardial infarction)	Hypertension/high blood pressure; diabetes type 2; atherosclerosis; arthritis; sciatica	Past medication for depression; current medication for anxiety; has never received psychological therapy	A=12 D=11	29
[illegible]21	Female	55-64	White British	None	Retired	Married	Acute coronary syndrome (myocardial	Hypertension; COPD; arthritis; duodenal ulcer; brittle bone	Past medication for depression; has received psychotherapy	A=9 D=5	9

							infarction)	disease; glaucoma; blood clotting; cataracts	previously		
[illegible]22	Male	55-64	White British	Not Collected	Not collected	Not collected	Not collected	Not collected	Not collected	Not collected	Not coll[illegible]
C23	Male	75+	White British	None	Retired	Married	Stable angina	Hypertension; high cholesterol; arthritis; hernia; diverticulitis	No history of relevant medication or psychological therapy	A=14 D=9	60
C24	Male	35-44	White British	None	Temporary sick leave	Single	Acute coronary syndrome (myocardial infarction)	High cholesterol; gout	No history of relevant medication or psychological therapy	A=11 D=8	58
[illegible]25	Male	35-44	White British	Degree	Unable to work due to long term disability or ill health	Divorced	Acute coronary syndrome (myocardial infarction)	Blood clotting;	No history of relevant medication or psychological therapy	A=15 D=12	24
[illegible]26	Male	35-44	White British	GCSE or equivalent	Unable to work due to long term disability or ill health	Divorced	Acute coronary syndrome (myocardial infarction)	Hypertension; high cholesterol; back pain	Currently medication for depression; has received psychotherapy previously	A=9 D=8	21
C27	Male	55-64	Asian British	None	Unable to work due to long term disability or ill health	Married	Acute coronary syndrome (myocardial infarction)	Diabetes type 2; high cholesterol; COPD; IBS or abdominal problems; kidney failure; cataracts	No history of relevant medication or psychological therapy	A=13 D=15	46

**28*** **	**Female**	**35-44**	**White British**	**GCSE or equivalent**	**Temporary sick leave**	**Cohabiting**	**Acute coronary syndrome (myocardial infarction)**	**Arthritis**	**No history of relevant medication or psychological therapy**	**A=12 D=11**	**43**
29	Male	45-54	White British	Vocational Qualification	Unable to work due to long term disability or ill health	Widowed	Adult congenital heart disease	COPD; arthritis	Past medication for depression and anxiety; has never received psychological therapy	A=11 D=10	55
C30	Female	65-74	White British	None	Retired	Widowed	Adult congenital heart disease	None	No history of relevant medication; has received bereavement counselling previously	A=5 D=10	16
31	Male	75+	White British	Vocational Qualification	Retired	Married	Atrial fibrillation	Hypertension; COPD	Current medication for depression and anxiety; has received counselling previously	A=10 D=6	11
32	Male	55-64	White British	None	Retired	Separated	Stable heart failure	IBS or abdominal problems; arthritis	Past medication for depression; has been treated by a mental health worker previously	A=11 D=11	24
33	Male	55-64	White	Vocational	In full time	Married	Acute	High cholesterol;	No history of	A=8	15

			British	Qualification	(paid) employment		coronary syndrome (myocardial infarction)	sleep apnoea	relevant medication or psychological therapy	D=6	
[illegible]34	Male	55-64	White British	None	In full time (paid) employment	Married	Acute coronary syndrome (myocardial infarction)	Atherosclerosis	Past medications for depression; has been treated by a psychiatrist previously	A=12<br>D=8	59
[illegible]35	Male	55-64	White British	A level	In full time (paid) employment	Divorced	Acute coronary syndrome (myocardial infarction)	COPD	No history of relevant medication or psychological therapy	A=8<br>D=2	7
[illegible]36	Male	65-74	White British	Not collected	Not collected	Not collected	Not collected	Not collected	Not collected	Not collected	Not coll[illegible]
[illegible]37	Male	35-44	White British	Postgraduate degree	In full time (paid) employment	Single	Acute coronary syndrome (myocardial infarction)	None	Current medication for depression; has received web-based CBT previously	A=9<br>D=3	11
[illegible]38	Female	55-64	White British	Diploma	Unemployed	Single	Stable heart failure	Arthritis	Past medications for depression and anxiety; has received CBT previously	A=8<br>D=3	41
[illegible]39	Female	75+	White British	Diploma	Retired	Married	Stable heart failure	Hypertension; high cholesterol; arthritis;	Current medication for depression and	A=12<br>D=16	27

								diverticilitis	anxiety; has received CBT and been treated by a psychiatrist previously		
40	Female	65-74	White British	Vocational Qualification	Retired	Divorced	Acute coronary syndrome (myocardial infarction)	Diabetes type 2	Current medication for depression; has received counselling previously	A=9 D=8	8
**41*** **	**Male**	**45-54**	**White British**	**GCSE or equivalent**	**Unable to work due to long term disability or ill health**	**Single**	**Acute coronary syndrome (myocardial infarction)**	**IBS; fabry disease; crohn's disease; renal failure**	**Past medication for depression and anxiety; has been treated by a psychologist previously**	**A=18 D=19**	**64**
42	Male	75+	White British	Diploma	Retired	Divorced	Acute coronary syndrome (myocardial infarction)	Hypertension; IBS; arthritis	Current medication for depression and anxiety; has received counselling previously	A=14 D=13	40
43	Male	55-64	White British	None	Unemployed	Single	Acute coronary syndrome (myocardial infarction)	None	No history of relevant medication or psychological therapy	A=9 D=9	58
44	Female	55-64	White British	Diploma	In part time (paid)	Divorced	Acute coronary	Hypertension; diabetes type 2;	Current medication for	A=15 D=3	27

					employment		syndrome (myocardial infarction)	arthritis; breast cancer	anxiety; has received CBT and counselling previously		
45	Male	45-54	White British	Vocational Qualification	Unable to work due to long term disability or ill health	Separated	Acute coronary syndrome (myocardial infarction)	High cholesterol	No history of relevant medication or psychological therapy	A=16 D=14	9
46	Male	55-64	White British	Vocational Qualification	In full time (paid) employment	Married	Acute coronary syndrome (myocardial infarction)	Breathing problems or COPD; IBS; arthritis	Past medication for depression; has received victim support counselling previously	A=9 D=6	30
**47*** **	**Female**	**45-54**	**White British**	**Diploma**	**Carer**	**Married**	**Stable angina**	**Hypertension**	**Current medication for depression; has never received psychological therapy**	**A=14 D=12**	**27**
48	Female	45-54	White British	None	Looking after family or home	Married	Acute coronary syndrome (myocardial infarction)	High cholesterol; Breathing problems or COPD	Current medication for depression and anxiety; has never received psychological therapy	A=10 D=8	68
49 237	Male	45-54	White British	Diploma	Temporary sick leave	Single	Acute coronary	High cholesterol; Ulcerative colitis;	Current medication for	A=13 D=9	29

							syndrome (myocardial infarction)	HIV	depression and anxiety; has received CBT previously		

Identification number: C = Control Arm; I – Intervention Arm; D = Declined to take part in the PATHWAY trial.

GCSE: General Certificate of Secondary Education; CBT: Cognitive Behavioural Therapy; COPD: Chronic Obstructive Pulmonary Disease; ME: Myalgic encephalomyelitis; IBS: Irritable bowel syndrome; MS: multiple sclerosis; HIV: Human Immunodeficiency Virus.

*HADS score: A = HADS Anxiety Sub-Scale Score; D = HADS Depression Sub-Scale Score. *Scores for both the anxiety and depression subscales of the HADS range from 0 to 21. Scores are categorised: 0-7 normal; 8-10 mild; 11-14 moderate; 15-21 severe (Zigmond and Snaith, 1983).

**Scores for the IES-R range from 0 to 88. Scores are categorised: 0-23 normal; 24-32 PTSD is a clinical concern; 33-88 probable PTSD diagnosis (Creamer et al., 2002; Weiss, 2007).

***Patients whose transcripts are presented in ‘Qualitative Analysis of Emotional Distress in Cardiac Patients from the Perspectives of Cognitive Behavioural and Metacognitive Theories: Why Might Cognitive Behavioural Therapy have Limited Benefit, and Might Metacognitive Therapy be More Effective?’
